# UV light-induced DNA lesions cause dissociation of yeast RNA polymerases-I and establishment of a specialized chromatin structure at rRNA genes

**DOI:** 10.1093/nar/gkt871

**Published:** 2013-10-04

**Authors:** Maxime Tremblay, Romain Charton, Manuel Wittner, Geneviève Levasseur, Joachim Griesenbeck, Antonio Conconi

**Affiliations:** ^1^Département de Microbiologie et Infectiologie, Faculté de Médecine, Université de Sherbrooke, Sherbrooke, QC J1E 4K8, Canada and ^2^Institut für Biochemie, Genetik und Mikrobiologie, Universität Regensburg, 93053 Regensburg, Germany

## Abstract

The cytotoxicity of UV light-induced DNA lesions results from their interference with transcription and replication. DNA lesions arrest elongating RNA polymerases, an event that triggers transcription-coupled nucleotide excision repair. Since arrested RNA polymerases reduce the accessibility of repair factors to DNA lesions, they might be displaced. The fate of arrested RNA polymerases-II at DNA lesions has been extensively studied, yielding partially contradictory results. Considerably less is known about RNA polymerases-I that transcribe nucleosomes-depleted rRNA genes at very high rate. To investigate the fate of arrested RNA polymerases-I at DNA lesions, chromatin-immunoprecipitation, electron microscopy, transcription run-on, psoralen-cross-linking and chromatin-endogenous cleavage were employed. We found that RNA polymerases-I density increased at the 5′-end of the gene, likely due to continued transcription initiation followed by elongation and pausing/release at the first DNA lesion. Most RNA polymerases-I dissociated downstream of the first DNA lesion, concomitant with chromatin closing that resulted from deposition of nucleosomes. Although nucleosomes were deposited, the high mobility group-box Hmo1 (component of actively transcribed rRNA genes) remained associated. After repair of DNA lesions, Hmo1 containing chromatin might help to restore transcription elongation and reopening of rRNA genes chromatin.

## INTRODUCTION

UV light-induced DNA lesions, like cyclobutane pyrimidine dimers (CPDs), are removed by nucleotide excision repair (NER). NER is subdivided into global genome repair (GGR), which repairs transcription inactive DNA and the nontranscribed strand (NTS) of transcribed genes, and transcription-coupled repair (TCR) that repairs the transcribed strand (TS) of transcribed genes only. In humans, the same 5 XP (xeroderma pigmentosum) gene products are required for both sub-pathways. In addition, GGR requires XPC and XPE, whereas TCR requires CSA and CSB (Cockayne syndrome proteins A and B). During NER: after DNA damage recognition, strand incisions on both sides of the damage and excision of a short strand containing the lesion, DNA synthesis takes place using the complementary DNA strand as template (1). CPDs in the TS block transcription and it is believed that arrested RNA polymerases-II (RNAPII) trigger TCR. Thus, the hallmark of TCR is fast removal of obstructions that impede elongation of RNA polymerases (2,3). The understanding of TCR in human has progressed considerably. Namely, arrested RNAPII signals the presence of DNA damage, recruiting the transcription-repair coupling factor (CSB) as well as the NER factors TFIIH, RPA, XPA, XPG and XPF (4). CSA and chromatin-associated factors also participate in TCR (5). After signaling the presence of DNA damage on the TS, arrested RNAPII might be displaced, a process that would provide access of NER factors to DNA lesions. One model proposes that RNAPII are released from the DNA and a second model that they are moved from the damaged site by reverse translocation (6). A third model suggests that an arrested RNAPII does not prevent the access of NER factors to the DNA lesion but that RNAPII could undergo conformational changes (7). Finally, a very low amount of RNAPII could bypass CPDs and the mechanism for this translesion was elucidated *in vitro* (8). Therefore despite the advanced knowledge on TCR, the outcome of RNAPII encountering DNA lesions is not clear. Even less is known about the fate of RNA polymerase-I (RNAPI) on damaged ribosomal genes (rRNA genes or rDNA).

Multiple copies of rRNA genes (∼150 in yeast) are organized in tandem repeats, of which only a fraction is transcribed. Inactive rRNA genes are assembled in nucleosomes, whereas active rRNA genes are largely depleted of nucleosomes (9–11) but densely loaded with RNAPI and high mobility group protein Hmo1 (12). The existence of two chromatin structures in the rDNA locus was demonstrated for a large variety of organisms, ranging from yeast to human (13), and rRNA synthesis is regulated by the transcription initiation rate, the number of active rRNA genes and, at least in human cells, by the elongation rate of RNAPI (10,14–17). Remarkably, after UV irradiation of yeast cells, transcription of rRNA genes stops (18). Here we addressed the fate of elongating RNAPI on the damaged TS and the rRNA gene chromatin during NER. Our findings revealed striking correlation between the presence of CPDs, block of transcription, dissociation of RNAPI and loading of histones downstream of the DNA lesion. Moreover, rRNA genes inactivated by UV irradiation adopted a specialized chromatin structure that was formed by nucleosomes but retained Hmo1. The protein Hmo1 is a marker of active rDNA chromatin and might help resumption of RNAPI transcription elongation and reopening of rRNA genes chromatin after DNA repair, which likely started at the transcription initiation site and then extended to downstream sequences.

## MATERIALS AND METHODS

### Strains, cells growth and UV irradiation

Yeast cells (Supplementary Table S1) were grown in complete medium (YEPD) to early log-phase (∼1.2 × 10^7^ cells/ml). Cultures were washed and resuspended in ice cold phosphate buffered saline, irradiated (254 nm) with a UV dose of 180 J/m^2^, harvested, resuspended in YEPD and incubated in the dark at 30°C with continuous shaking for different repair times.

### Nuclei isolation, psoralen cross-linking, chromatin immunoprecipitation and chromatin endogenous cleavage

Nuclei isolation and psoralen cross-linking were done as described in (19), and chromatin immunoprecipitation (ChIP) was done mostly as described in (20): 1.4 ml of 37% formaldehyde were added to 50 ml of cells suspension and incubated at growing temperature for 20 min. After quenching formaldehyde with 125 mM glycine at room temperature for 5 min, cells were collected, washed three times in ice cold TBS (20 mM Tris–HCl, pH 8, 150 mM NaCl) and then the pellets were frozen in liquid nitrogen. Thereafter, the pellets were resuspended in 700 μl of Lysis buffer (50 mM HEPES, pH 7.5, 140 mM NaCl, 1 mM ethylenediaminetetraacetic acid (EDTA), 1% Triton X-100, 0.1% Na-Deoxycholate, 1 mM PMSF, 1 M benzamidine, 10 mg/ml aprotinin, 1 mg/ml leupeptin, 1 mg/ml pepstatin), ruptured by adding 400 μl of glass beads (425–600 μm; Sigma) and vortexed at maximum speed for 2 h at 4°C. Cell lysates were separated from the glass beads and their volumes adjusted to 1.1 ml with Lysis buffer. Shearing of chromatin was done by sonication. From each sample, 5 μl aliquots labeled whole cell extract (WCE) were stored at −20°C. In parallel, 500 μl of WCE were mixed either with with 30 μl of Protein-A sepharose beads (CL-4B; GE Healthcare) coated with rabbit polyclonal antibodies (ab9110, Abcam) to detect HA tagged proteins, or with IgG sepharose beads (GE Healthcare) to detect TAP tagged proteins. After overnight incubation with continuous rotation at 4°C and centrifugation (500×g, 1 min, 4°C), the pellets were washed two times in Lysis buffer without protease inhibitors, two times with Lysis buffer plus 360 mM NaCl, two times with Wash buffer (10 mM Tris–HCl, pH 8.0, 250 mM LiCl, 0.5% NP40, 0.5% Na-deoxycholate, 1 mM EDTA) and one time with TE buffer. The final pellets were resuspended in 100 μl of Elution buffer (50 mM Tris–HCl, pH 8.0, 10 mM EDTA, 1% sodium dodecyl sulfate (SDS)) and incubated at 65°C for 10 min. After centrifugation, 90 µl of each supernatant were collected and 100 μl of TE/SDS (10 mM Tris–HCl, pH 8.0, 1 mM EDTA, 1% SDS) were added to the remaining beads, followed by incubation at 65°C for 10 min. After centrifugation, 90 µl of each supernatant were collected and added to the first collected 90 µl, together with an additional 120 μl of TE/SDS (total 300 µl). In parallel, 95 μl of TE/SDS were added to the 5 μl aliquots of WCE. The samples were incubated overnight at 65°C. The following day, 140 μl TE were added, together with 3 μl of glycogen (Roche, Mannheim, Germany) and 2.5 μl of RNAse A (10 μg/μl). After 1 h incubation at 37°C, 7.5 μl of Proteinase K (20 mg/ml) were added followed by an additional 1 h incubation at 37°C. DNA was purified by phenol extraction and precipitated in ethanol. The resuspended DNA was analyzed by quantitative polymerase chain reaction (qPCR) as recommended by the manufacturer. We note that DNA was not repaired prior to qPCR analyses, since the DNA was sheared to small fragments that, on average, did not have enough DNA damage to influence the results by blocking Taq polymerases. The computed tomography values confirmed that similar amounts of DNA were synthesized in the input fractions prior and soon after UV irradiation, in both *WT* and *rad14Δ* strains.

Chromatin endogenous cleavage (ChEC)-psoralen was done as described in (12). Briefly, 80 µl of nuclei suspensions isolated from formaldehyde-cross-linked cells (∼2.8 × 10^8^) were mock treated or incubated with CaCl_2_ (2 mM final concentration) for the indicated times at 30°C. The reactions were stopped by addition of 100 µl of IRN buffer (50 mM Tris–HCl, pH 8, 20 mM EDTA and 0.6 M NaCl).

### Miller spreading

Yeast was prepared as in (21). After Zymolyase treatment in 1 M Sorbitol and 250 mM EDTA (pH 8.0) for 4 min at 30°C, cells were collected, 1 ml Triton X-100 (0.025%, pH 9.0) was added, and incubated for 5 min at room temperature. Electron microscopy grids (CF300-Cu, Cedarlane) were dehydrated in 100% ethanol, rinsed in sucrose-formaldehyde (0.1 M sucrose, 10% v/v formaldehyde, pH 8.7), placed at the bottom of centrifugation chambers and covered with a sucrose-formaldehyde cushion. In all, 7 µl of spheroplasts were added to the cushion and centrifuged. Grids were washed as in (21) and analyzed with a Hitachi H-7500 TEM.

### Transcription run-on

A total of 100 µl of nuclei in NIB buffer (∼2 × 10^8^ cells) were added to 100 µl of TRO reaction mixture (18). Samples were held on ice for 5 min before transcription run-on (TRO) was initiated at 25°C and allowed for 10 min. RNA was isolated and quantified. rDNA fragments (2 µg each) were immobilized on nylon membrane and hybridized to the radiolabeled RNA. Phosphorimager signals were quantified (ImageQuaNT, GE-Healthcare).

## RESULTS

### Presence of CPDs correlates with RNAPI dissociation

Although studies using *in vivo* models have to compromise with populations of randomly damaged DNA, the calculation for Gaussian distributions of CPDs in sequential fragments of the rDNA coding region allowed to determine the average length, from the transcription start site, where most of the rRNA genes had at least one CPD (Supplementary Figure S1). We defined such DNA region as ‘the likely position of the first CPD’, which corresponds to ∼2.96 kb downstream of the transcription start site and where most rRNA genes (∼87.3%) have at least 1 CPD in the TS. DNA length affects the frequency at which CPDs form and only ∼37.9% of the rRNA genes had at least 1 CPD in the 0.86 kb fragment. Previously we measured DNA repair in the rDNA locus (18,22), and the results are reported here as references: between ∼40 and ∼60% of CPDs were repaired within the first 30 min, and between ∼60 and ∼90% of CPDs were repaired between 1 and 4 h after irradiation.

**Figure 1.**
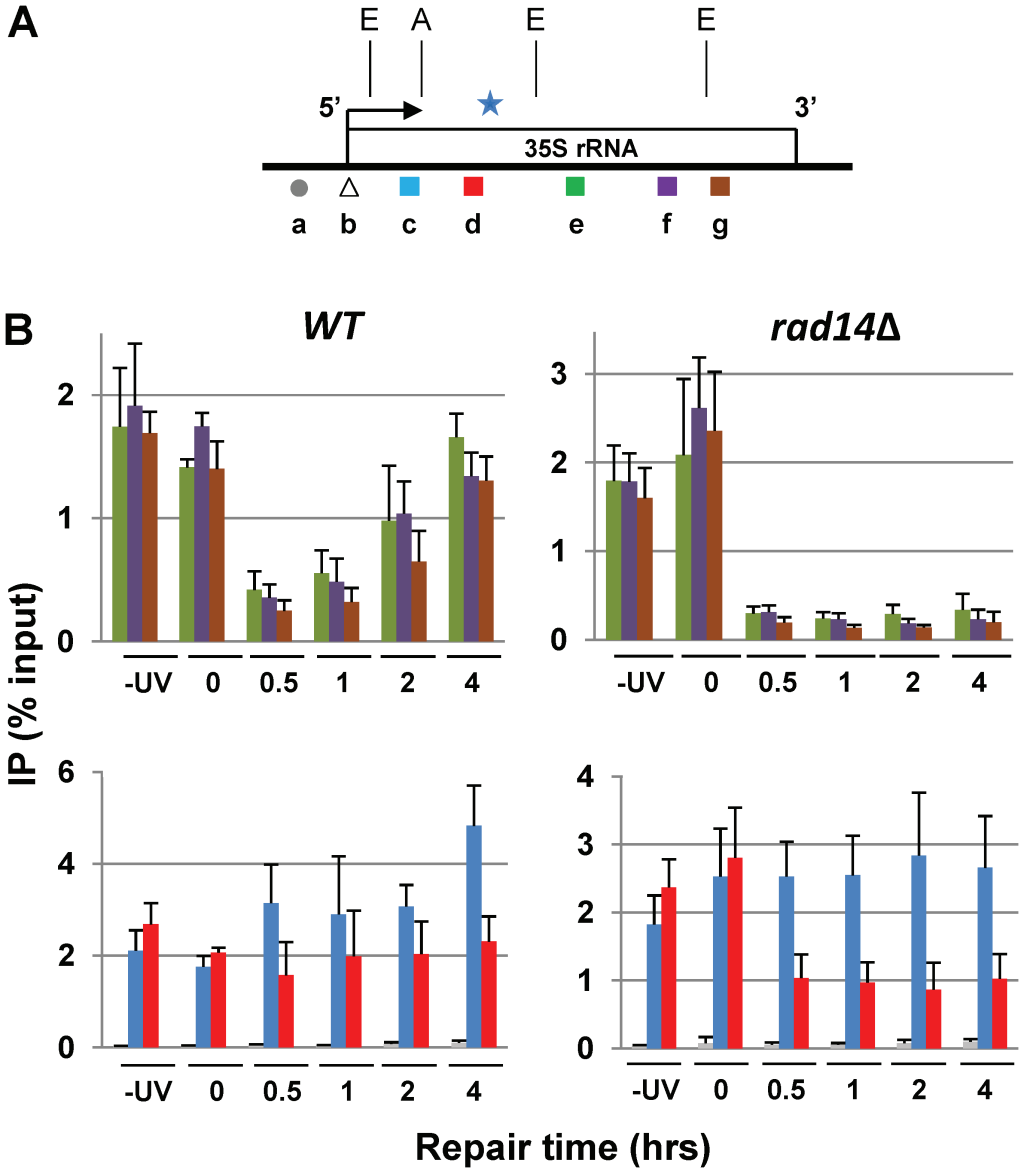
Dissociation of RNAPI from UV damaged TS. (**A**) Map of 35S rRNA gene; 5′-, 3′-end and direction of transcription (arrow) are shown. Amplicons ‘a’ to ‘g’ (average length ∼150 bp) synthesized in real-time polymerase chain reaction (qPCR); ‘b’ is used for the experiment described in Figure 2B. Star indicates the approximate ∼2.96 kb region downstream of transcription start site (additional information is presented in Supplementary Figure S1). (**B**) ChIP. Upper panel; data for amplicons ‘e’, ‘f’ and ‘g’. Lower panel; data for amplicons ‘a’ (control), ‘c’ and ‘d’. Means (±1 SD) of four independent biological experiments represent percent of input of total cellular extract.

To determine the fate of RNAPI on damaged rDNA, the occupancy of RNAPI was mapped throughout the rDNA-coding sequence: before irradiation, soon after and during repair. ChIP on formaldehyde cross-linked cells was performed with IgG-coated beads that bind TAP tagged A190 subunit of RNAPI, and real-time amplification of coprecipitated DNA was done as detailed in ‘Materials and Methods’ section, using a series of amplicons that span the rDNA unit (Figure 1A). The data obtained for each immunoprecipitation are shown in Figure 1B. In *WT* (*NER^+^*) cells, drop in RNAPI occupancy was observed in the central and 3′-portions of rRNA genes, between 0.5 and 1 h after irradiation (*WT*; amplicons-e, -f, -g). After 2 h, there was ∼50% recovery and at 4 h most of the RNAPI occupancy was restored. A different result was found for the *rad14Δ* cells (*NER^_^*) in which CPDs were not removed from the rDNA locus, and most RNAPI definitively dissociated from the central and 3′-terminal portions of the coding region (*rad14Δ*; amplicons-d, -e, -f, -g). Conversely, in both *WT* and *rad14Δ* cells RNAPI accumulated (about 1.5–2 times) at the 5′-end of rRNA genes throughout the incubation time (*WT*; amplicons-c, -d and *rad14Δ*; amplicon-c). Amplicon-a, which is outside of the rDNA-coding region, was used as control to determine the background level of ChIP. The results indicate that the DNA region spanning the start of transcription and the likely position of the first CPD was densely packed with RNAPI. In contrast, RNAPI density decreased in the central and 3′ terminal DNA regions, beyond the region that statistically carried the likely position of the first CPD (Figure 1 and Supplementary Figure S1; amplicon-d, ∼2.96 kb downstream of the transcription start site).

### RNAPI stability and pre-initiation complex formation in UV-irradiated cells

It was reported that when DNA lesions persist stalled RNAPII are removed from DNA damage sites by the proteasome (23). The results presented here showed depletion of RNAPI in the central and 3′ terminal rDNA regions (Figure 1B; upper panel) but continuous association of RNAPI within the rDNA 5′-end region (Figure 1B, lower panel), suggesting that in both *WT* and *rad14Δ* cells there was a significant pool of endogenous RNAPI (24) even after UV irradiation. Nevertheless, we considered the possibility that the proteasome was involved in the displacement of RNAPI and analyzed its presence on damaged rDNA in temperature sensitive mutants for the 19S regulatory subunit of the proteasome. The results shown in Supplementary Figure S2 indicate that an active proteasome is not required for the displacement of RNAPI from the UV-damaged TS.

**Figure 2.**
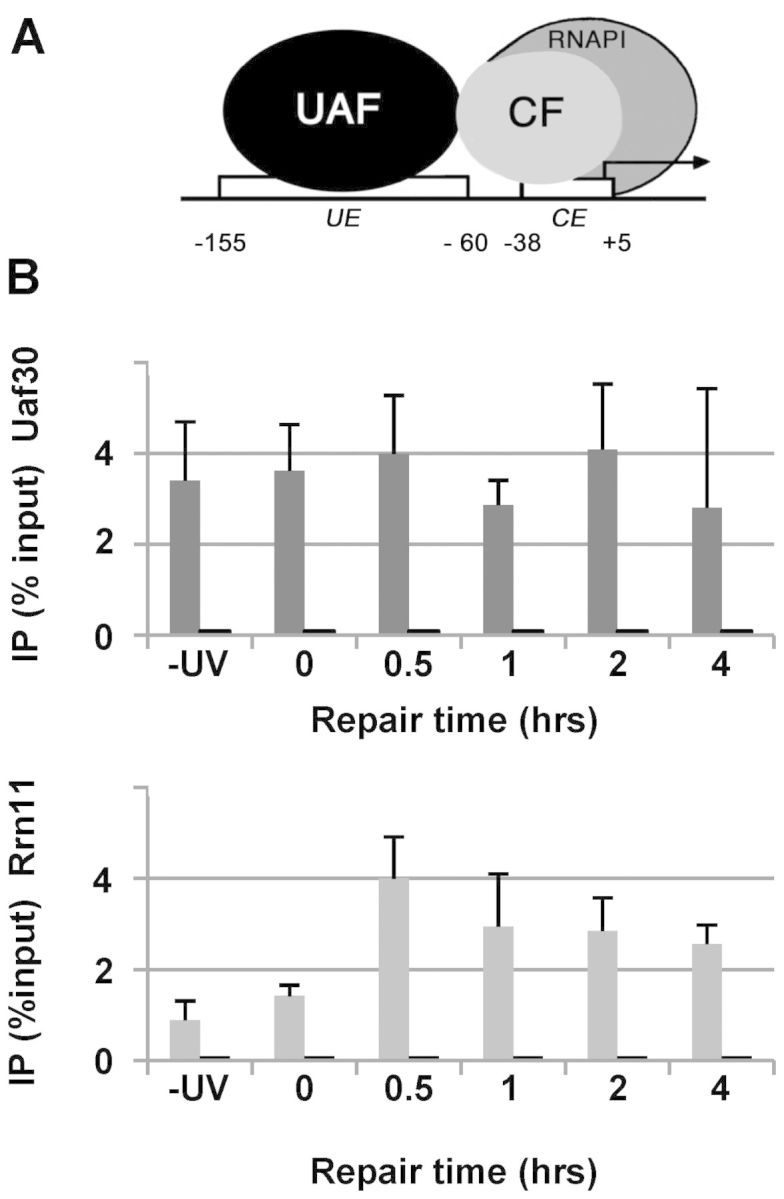
(**A**) rDNA promoter; UE, CE, UAF and CF. (**B**) ChIP; amplicon ‘b’ (Figure 1A) is a qPCR 113 bp fragment (gray bars), amplicon ‘e’ (Figure 1A) was used as control (short black bars). Means (±1 SD) of four independent biological experiments represent the percent of input of total cellular extract.

In yeast, initiation of transcription requires the assembly of the upstream activating factor (UAF; formed by Uaf30, Rrn5, Rrn9, Rrn10, H3 and H4) on the upstream element (UE) and of the core factor (CF; formed by Rrn6, Rrn7 and Rrn11) on the core element (CE) (Figure 2A). However, whereas UAF binds to promoters of both inactive and active rRNA genes, CF mostly binds to the promoters of active rRNA genes (25,26). To define the presence of RNAPI pre-initiation complex after UV irradiation, the association of HA tagged UAF and CF with the rDNA promoter of *WT* (*NER^+^*) cells was analyzed by ChIP. The real-time amplification of co-precipitated DNA was done using amplicon-b in the promoter and amplicon-e as control (Figure 1A). The data obtained for each immunoprecipitation are shown in Figure 2B. Association of UAF with the rDNA promoter was similar before UV irradiation and throughout the repair times (upper panel), whereas the association of CF increased ∼3 times, on average, after irradiation (lower panel). Thus increase in binding of CF at the rDNA promoter closely corroborates the ∼1.5 to 2 times increase in RNAPI at the 5′-end after UV irradiation (Figure 1B; lower panel).

### Arrangements of rDNA/RNAPI/rRNA ternary complexes during NER

**Figure 3.**
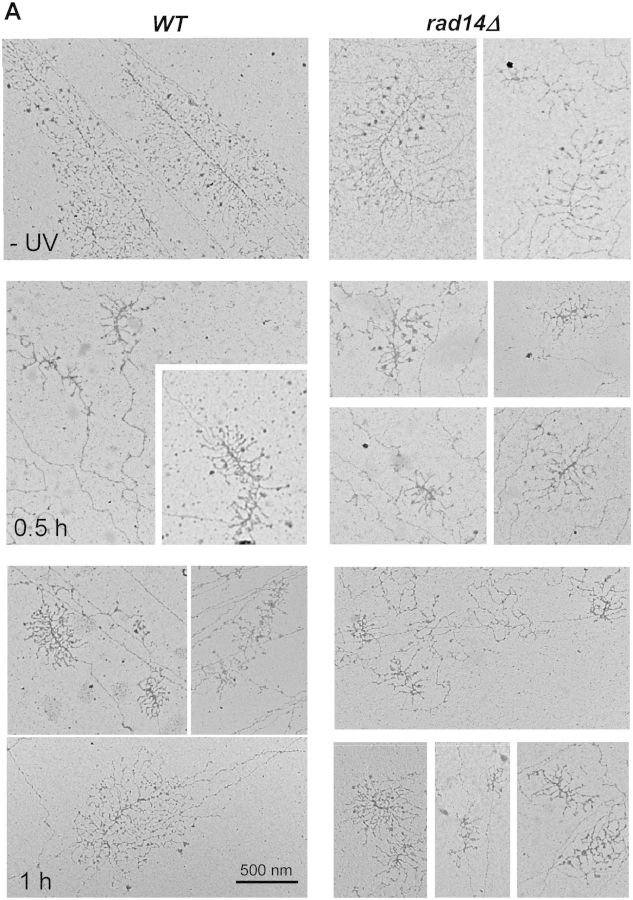

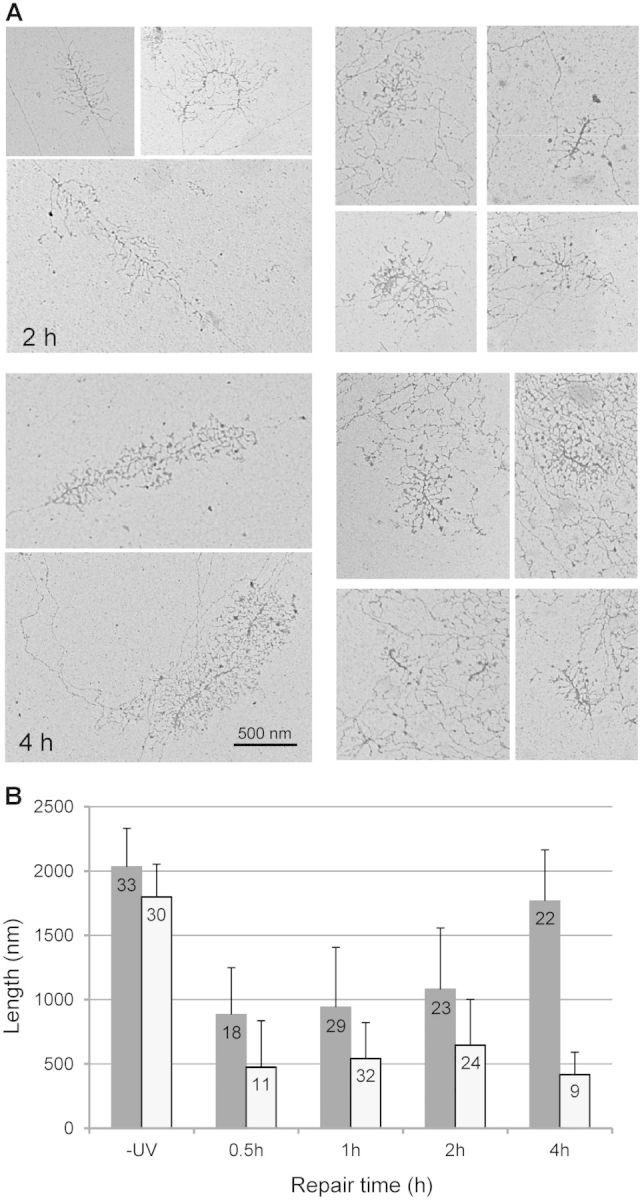
Electron micrographs of rRNA genes taken at different times after irradiation. (**A**) Chromatin spreads of log phase cells before irradiation (−UV) and at different times during repair (0.5–4 h), for wild-type (*WT RAD14*) and *rad14Δ* cells. (B): After visualization of rDNA transcription units from *WT* and *rad14Δ*, lengths were measured with ImageJ software (http://rsbweb.nih.gov/ij/) and compared to internal scale bars (100 and 500 nm). Data were from different preparations of chromatin spreads. The numbers of transcription units analyzed are described in the histogram and are shown in Supplementary Figure S3. Average lengths are in nm (SE: standard errors). *WT*; gray columns and *rad14Δ*; white columns.

A hallmark for RNAPI transcription is the rDNA/RNAPI/rRNA ternary complex forming the characteristic Christmas tree-like structure. Hence, chromatin was spread onto electron microscopy grids (21) to visualize the ternary complexes and to measure their lengths during NER, in *WT* (*RAD14*) and in *rad14Δ* cells (Figure 3A). Before irradiation (−UV), micrographs obtained from both strains were very similar. For the *WT*, after 0.5 h repair micrographs showed a mixed population of short transcription units, the lengths of which gradually increased as repair proceeded (1–4 h). For the *rad14Δ*, micrographs showed short transcription units throughout the entire incubation time and full-length units were practically not found. Already after 0.5 h, the transcription units in *WT* cells were, on average, longer than those observed in *rad14Δ* cells reflecting the efficiency of NER in the *WT* (Figure 3B; histogram of average lengths. Single measurements of transcription units are shown in Supplementary Figure S3). Consistent with the results obtained by ChIP of RNAPI, chromatin spreads for the *WT* showed that recovery of full-length ternary complexes was almost complete after 4 h repair. In *rad14Δ*, the ternary complexes were detectable only at the estimated rDNA 5′-region throughout the incubation time after UV irradiation, also correlating with the results obtained by ChIP. These results suggested that RNAPI elongated from the promoter to the first DNA lesion on the TS.

**Figure 4.**
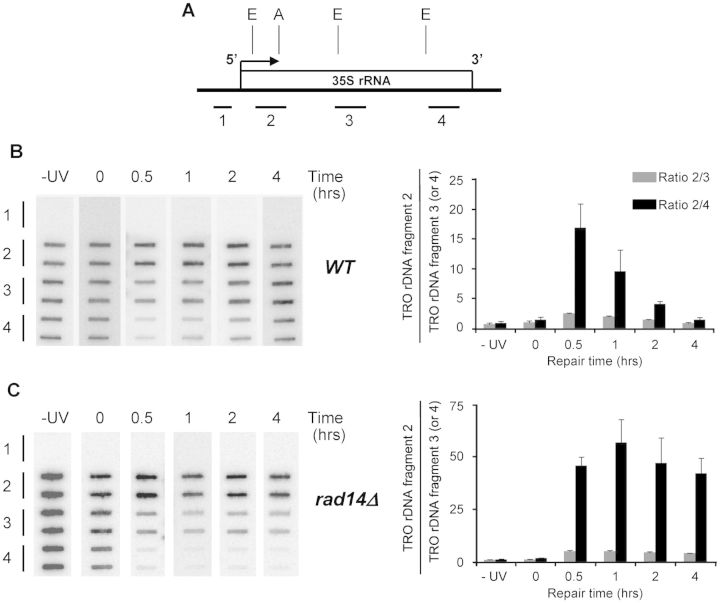
RNAPI elongation in wild-type (*WT RAD14*) and *rad14Δ* cells before and at different times after UV irradiation, at the 5′-end, middle and 3′-end of rRNA gene. (**A**) EcoRI (E) and ApaI (A) are shown as references to Supplementary Figure S1 and Figures 1A and 5A. Bars position the rDNA fragments: 1; 532 bp, 2 to 4; average length of ∼950 bp. (**B**) Left panel; nuclei from nonirradiated (−UV) and irradiated (0, 0.5, 1, 2 and 4 h) *WT* were incubated in TRO reaction conditions containing [α-^32^P]UTP. Purified elongated, radio-labeled RNAs were used as probes to hybridize the membrane-bound rDNA fragments (1–4), spotted in duplicate onto filter membranes. Fragment 1 was the control for hybridization specificity. Right panel; quantification of phosphoimager signals. Histogram shows measurements for the 5′-end (rDNA-fragment 2) either normalized to the corresponding measurements for the middle (rDNA-fragment 3, gray bars) or the 3′-end (rDNA-fragment 4, black bars). Means (±1 SD) of three independent biological experiments. (**C**): Same as in (B) for *rad14Δ*.

To test if the ternary complexes observed in chromatin spreads were not stalled but indeed elongating, the TRO assay was applied. TRO uses endogenous chromatin as template for transcription and, therefore, allows monitoring elongation of RNA polymerases that were transcribing prior to nuclei isolation (27). Here, RNAPI elongation measured by TRO reflected RNAPI activity in intact cells, before and after UV irradiation and during NER. Four different rDNA fragments, spanning the entire rRNA gene (Figure 4A), were spotted onto filter membranes and hybridized with radiolabeled RNA obtained from TRO reactions (Figures 4B and C, left panels). Quantification of signals (right panels) showed that before UV irradiation (−UV) and soon after (0 h), in *WT* (*RAD14*) and *rad14Δ*, RNAPI elongation (or rRNA production) was similar at the 5′-end, in the middle and at the 3′-end of the gene. Namely, the TRO ratios between rDNA fragment 2 and rDNA fragment 3 or rDNA fragment 4 gave values that were near to 1 (e.g. for −UV; *WT*: 0.7 ± 0.2; 0.9 ± 0.3 and *rad14Δ*: 0.9 ± 0.2; 1.1 ± 0.3 for the 5′-end to middle and 5′-end to 3′-end ratios, respectively). These results indicated that before irradiation RNAPI were distributed throughout the rDNA locus. In contrary, after 0.5 h ∼3.8 times more rRNA was produced at the 5′-end than in the middle of the gene, and considerably more rRNA was produced at the 5′-end than at the 3′-end. Measurements for the *WT* showed that the TRO ratios gradually returned to ∼1 after 4 h incubation, whereas measurements of RNAPI elongation for the *rad14Δ* remained higher at the 5′-end than in the middle and at the 3′-end of the gene, for the entire incubation time. Already after 0.5 h incubation, the 5′-end to 3′-end ratios were smaller for the *WT* than for the *rad14Δ* strain, which reflected the DNA repair kinetics (18). These data corroborated the presence of RNAPI measured by ChIP (Figure 1B) and the difference in average lengths of the ternary complexes (Figure 3B). Moreover, they imply that RNAPI that were associated with rDNA between the 5′-end and the likely position of the first CPD were transcription competent.

### Changes in rDNA chromatin after UV irradiation

**Figure 5.**
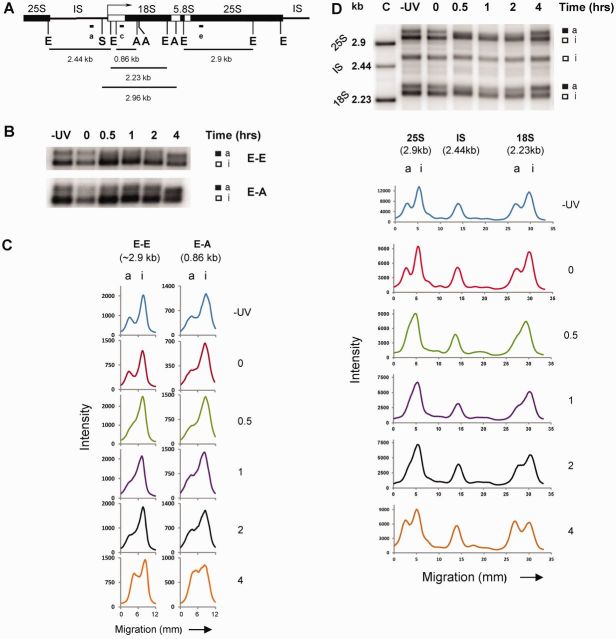
Chromatin structure of rRNA genes during NER. (**A**) IS, 18S, 5.8S and 25S are the intergenic spacer and the three regions of the coding portion of rDNA. ‘a’, ‘c’ and ‘e’ represent the probes (same as amplicons in Figure 1A), E (EcoRI), S (SmaI) and A (ApaI). (**B**) Nuclei from *WT* (*RAD14*) cells collected before UV irradiation (−UV), soon after (0) and at different repair times (0.5, 1, 2 and 4 h), were cross-linked with psoralen. DNA was extracted and digested with the respective restriction enzymes and separated on 1% native agarose gels. After blotting, filter membranes were hybridized with ^32^P end-labeled oligonucleotide ‘e’ or ‘c’; a: active and i: inactive rDNA chromatin. (**C**) Scan profiles of gels shown in (B). (**D**) Upper panel; DNA was extracted from noncross-linked (control, C) and psoralen cross-linked nuclei (−UV to 4 h), and digested with EcoRI. Filter membranes were hybridized with the combination of ^32^P end-labeled oligonucleotides ‘a’, ‘c’ and ‘e’. Lower panel; scan profiles of the gel shown.

Psoralen cross-linking is used to measure the proportion of open (or active) versus closed (or inactive) rRNA gene chromatin. Open rDNA chromatin binds more psoralen than closed rDNA chromatin, and purified psoralen cross-linked DNA from the former fraction migrates slower in native agarose gel electrophoresis than psoralen cross-linked DNA isolated from the latter fraction (reviewed in 13). Psoralen cross-linking was used to monitor the structure of rDNA chromatin during NER. *WT* (*NER^+^*) TAP tagged cells were collected before UV irradiation, soon after and during repair. Nuclei were isolated and treated with psoralen. Following DNA purification and restriction enzyme digestion, the fragmented DNA was separated by gel electrophoresis and blotted. Changes in rDNA chromatin during NER were measured in the central portion and at the 5′-end of the gene (Figure 5A; E-E, 2.9 kb and E-A, 0.86 kb, respectively). Before UV irradiation and immediately after, rRNA genes were separated into open and closed copies (Figure 5B and C, −UV and 0; a- and i- band, respectively). During the first hour after irradiation, the central portion of rDNA underwent changes in psoralen accessibility indicating gradual, but not complete, closing of active rRNA genes (E-E; transition of the a-band into a smear above the i-band). At 2 h, there was some recovery of the active copies and at 4 h an even higher percentage of open rRNA genes than before UV irradiation was observed (Figure 5C; E-E, 4 h). During the same repair times, the 5′-end region largely retained the open chromatin structure, although some chromatin rearrangement occurred since ∼37.9% of rRNA genes had at least 1 CPD in the 0.86 kb EcoRI–ApaI fragment, which promoted block of RNAPI and chromatin inactivation (Figure 5C; E-A, 0.5 h). Analyses at the 5′-end also showed additional opening of rRNA genes at 4 h after irradiation (E-A, 4 h). Taken together, these data are consistent with a model where chromatin is kept open between the promoter and the likely position of the first CPD by RNAPI transcription. Beyond the likely position of the first CPD most RNA polymerases are displaced, concomitantly with chromatin closing.

These results show that after 4 h repair, more than the original percent of open rRNA genes was restored. Since it was recently demonstrated that most of the rRNA genes assumed an open chromatin conformation when DNA replication was prevented and cells were arrested in the G_1_-phase of the cell cycle by α-factor (11), and that DNA lesions halt the progression of the replication fork (28), we followed cycle progressions at different times after UV irradiation by flow cytometry. The results presented in Supplementary Figure S4 indicate that at 4 h after irradiation there was partial accumulation of cells in G_1_/S, which could explain the opening of additional rDNA transcription units (Figure 5C, 4 h).

### Reopening of rDNA chromatin starts at the 5′-end

The data obtained by ChIP, electron microscopy and TRO suggested that reopening of chromatin could occur in a 5′- to 3′-end direction. Thus, psoralen cross-linking was applied to compare the temporal rearrangement of rDNA chromatin during NER in three different EcoRI fragments (Figure 5A): the 2.44 kb E-E fragment comprising the entire intergenic spacer (IS) between rRNA genes that is covered by nucleosomes (control); the 2.23 kb E-E fragment containing most of the 18S region; and the 2.9 kb E-E fragment containing a large portion of the 25S region. UV irradiation of *WT* (*NER^+^*) cells, psoralen cross-linking and DNA analyses by agarose gel electrophoresis were done as described above. Filter membranes were hybridized with the 3′-end-labeled oligonucleotides ‘a’, ‘c’ and ‘e’ simultaneously and the results are shown in Figure 5D. Samples of DNA isolated from uncross-linked nuclei that were used as controls (upper panel; Figure 5C) showed the three E-E fragments corresponding to the 25S, IS and 18S regions. After psoralen treatment, the same rDNA fragments showed the characteristic slow migration caused by psoralen cross-linking (−UV) (e.g., 10). As expected, the doublet of bands (active and inactive rDNA chromatin) was observed for the 25S and 18S rRNA coding fragments but not for the IS fragment. Moreover, changes in rDNA chromatin during NER were observed for the coding regions only and were as described in Figure 5B and C (E-E fragment). Remarkably, the data showed that reappearance of the a-band during repair happened earlier in the 18S than in the 25S region (lower panel). These results suggest that after removal of CPDs, opening of rDNA chromatin was re-established starting at the 5′-end and sequentially expanding to the downstream portion of the gene. Again, after 4 h we observed a higher percentage of open rRNA genes than before UV irradiation.

### rDNA chromatin composition after UV irradiation and during NER

ChIP could not be used to characterize the composition of active and inactive rDNA (e.g.; presence, or absence, of histone and other proteins) because the coexistence of distinct rDNA chromatin populations limits the interpretation of the data. In fact, determining the amount of histones associated with the rDNA locus is compromised because the results represent only an average of active- and inactive-rDNA copies. Thus, psoralen cross-linking and ChEC were combined to overcome the limitations of ChIP when applied to rDNA chromatin. ChEC is a very sensitive assay for assessing the presence and position of DNA-binding proteins *in vivo* (29). It is based on the tagging of C-termini of endogenous proteins with micrococcal nuclease. *In vivo*, the nuclease is inactive since the concentration of Ca^2+^ is low (50–200 nM). Upon exogenous addition of Ca^2+^, the micrococcal nuclease is activated and introduces strand breaks within ∼100 to 200 bp of the DNA-binding site of the tagged protein. Consequently, mapping of strand breaks determines the position of the tagged protein on the DNA. To partially characterize the composition of rDNA chromatin during NER, namely the a-band, i-band and the smear between the two rDNA populations, psoralen cross-linking and ChEC were combined. The induction of micrococcal nuclease caused selective degradation (or decrease in signal intensity) of the rDNA population that contained the tagged protein (see Supplementary Figure S5 for additional information).

**Figure 6.**
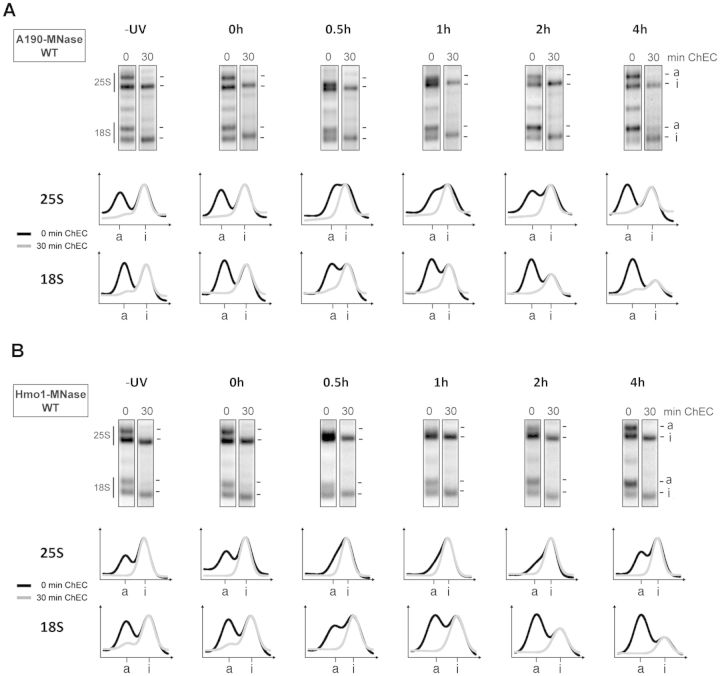

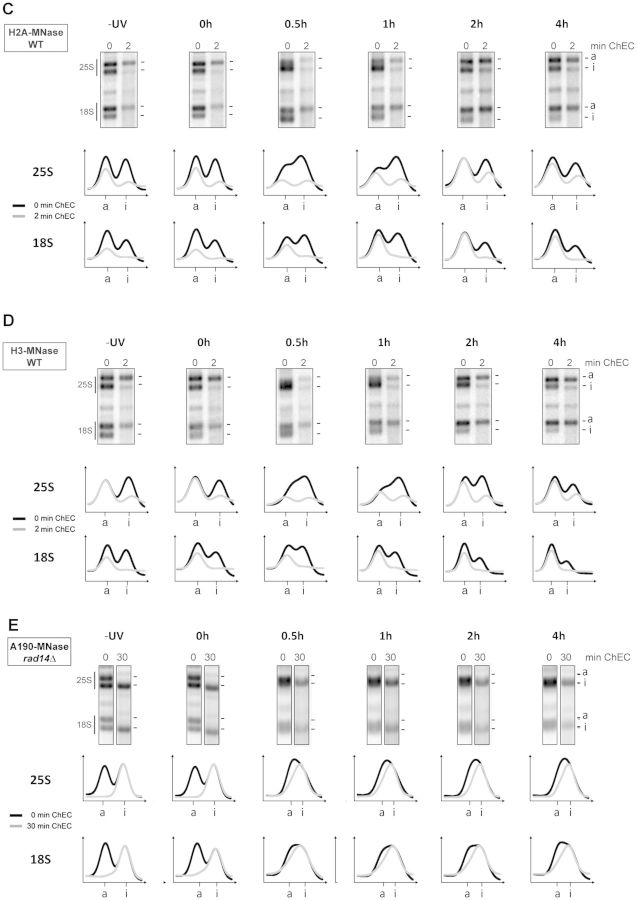

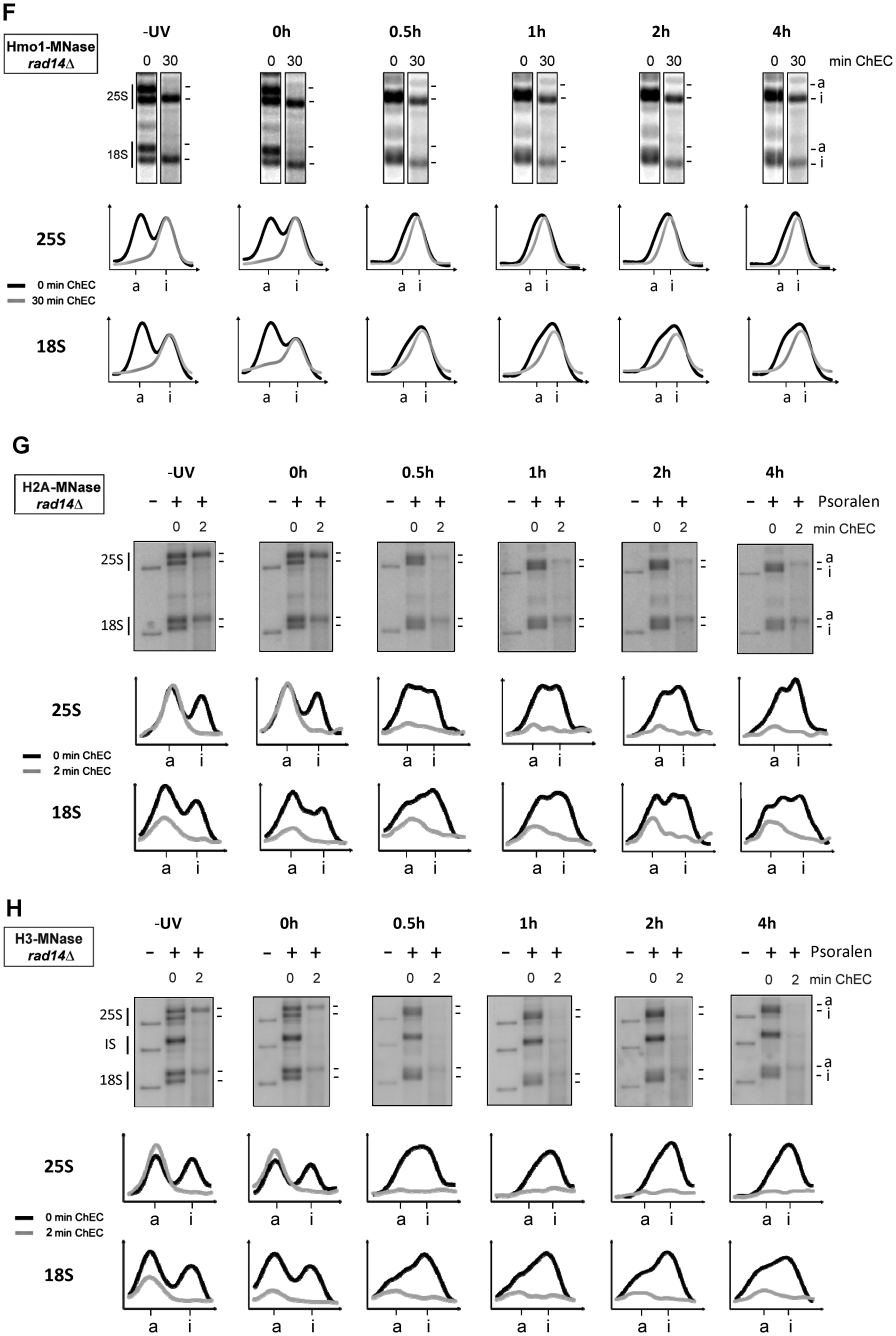
Closing and reopening of rRNA genes chromatin resulted from nucleosomes loading and unloading. (**A–D**) Nuclei were isolated from *WT* (*RAD14*) expressing RNAPI subunit A190-, Hmo1-, H2A- or H3-micrococcal nuclease fusion proteins, before (−UV) and after UV irradiation (0–4 h). ChEC was initiated by adding CaCl_2_ to nuclei suspensions and DNA cleavage allowed for various times (indicated by ‘min ChEC’, see Supplementary Figure S6A–D), where ‘0’ is the control (CaCl_2_ mock treated). Only 30 and 2 min nuclease digestions are shown in A, B and C, D, respectively. After addition of EDTA, psoralen cross-linking, DNA extraction and EcoRI digestion, filter membranes were probed to detect the 2.23 and 2.9 kb EcoRI fragments, containing portions of the 18S and 25S regions (Figure 5A). Upper panels; data obtained from yeast strains carrying (A) A190-, (B) Hmo1-, (C) H2A- and (D) H3- micrococcal nuclease fused protein. ‘a’ active and ‘i’ inactive rDNA chromatin. Lower panels; scan profiles. Signal intensities of A190 and Hmo1 micrococcal nuclease fusion proteins were normalized to peak values and plotted against the gel migration. Signal intensities of histone micrococcal nuclease fusion proteins were not normalized. (**E–H**): Nuclei were isolated from *rad14Δ* cells expressing the RNAPI subunit A190-, Hmo1-, H2A- or H3-micrococcal nuclease fusion proteins. Experiments were as done as described above and ChEC entire time courses are in Supplementary Figures S6E–H. Figure labeling is as in (6 A to D) but control lanes for psoralen cross-linking (no psoralen; −) were added in (G) and (H). In (H), filter membranes were hybridized with the same combination of probes used for experiments shown in Figure 5D to detect the restriction fragments for the 25S, IS and 18S.

*WT* (*NER^+^*) cells carrying micrococcal nuclease-tagged RNAPI subunit 190 or Hmo1 (components of active rRNA genes) and histone H2A or H3 (components of inactive rRNA genes), were not treated or treated with UV light and incubated thereafter for the indicated times. Nuclei were isolated, Ca^2+^ was added to induce micrococcal nuclease cleavage and incubated for different times. DNA cleavage was stopped and the nuclei were cross-linked with psoralen, DNA was isolated, digested with EcoRI (see Figure 5A; 18S and 25S; 2.23 and 2.9 kb fragments, respectively) and analyzed by agarose gel electrophoresis. The results for a single time point of the micrococcal nuclease digestion are presented in Figure 6A–D and the whole ChEC time courses are in Supplementary Figure S6A–D. As shown in Figure 5 for yeast carrying TAP tagged RNAPI, before UV irradiation and immediately after, rRNA genes were separated into active and inactive copies (0 min ChEC, −UV and 0 h). During the first hour after irradiation, changes in psoralen accessibility indicated a gradual, although not complete, closing of the active rRNA genes (25S: 0 min ChEC, 0.5 and 1 h; 18S: 0.5 h). For the 25S region, reopening occurred 2 h after irradiation (25S: 0 min ChEC, 2 h). For the 18S region reopening of rRNA genes occurred already between 0.5 and 1 h after irradiation, again implying that reopening of chromatin started in the 18S region (18S: 0 min ChEC, 0.5 h and 1 h). After activation of micrococcal nuclease fused to A190 and Hmo1 proteins (6 A and B, respectively), before and after irradiation (−UV, 0 h) the a-band was preferentially degraded (compare 0 and 30 min ChEC), indicating that RNAPI and Hmo1 were components of the active rRNA gene chromatin. Moreover, the smear between the a- and i-bands (25S: 0.5 and 1 h; 18S: 0.5 h) was also degraded implying that the two markers for active rRNA genes were present in this rDNA subpopulation. Following reopening of rRNA genes, only the a-band was degraded (25S: 2 h and 4 h; 18S: 1 h to 4 h). After activation of the micrococcal nuclease fused to H2A and H3 proteins (6 C and D, respectively), before and after irradiation (−UV, 0 h) the i-band was preferentially degraded (compare 0 and 2 min), indicating that nucleosomes were components of the inactive rRNA genes chromatin. The smear between the a- and i-bands (25S: 0.5 h and 1 h; 18S: 0.5 h) was degraded, together with the i-band. The results suggest that the subpopulation of rDNA chromatin, migrating between the inactive and active rRNA genes chromatin, was partially composed of RNAPI, Hmo1 and nucleosomes.

In parallel, the same set of proteins was tagged in the NER-deficient *rad14Δ* cells. The results for a single time point of the micrococcal nuclease digestion are presented in Figures 6E–H and the whole ChEC time courses are in Supplementary Figure S6E–H. Before UV irradiation and immediately after, rRNA genes were separated into active and inactive copies (0 min ChEC, −UV and 0 h). Thereafter, the a-band changed into a smear but, in contrast to *WT*, there was no reopening of rRNA gene chromatin even after 4 h incubation (0 min ChEC, 0.5–4 h). The results obtained upon activation of micrococcal nuclease fused to A190 and Hmo1 proteins, or to H2A and H3 proteins, before and immediately after irradiation, were very similar to those obtained for the corresponding *WT* (*NER^+^*) strains. Conversely, however, at later times after UV irradiation the degradation of rDNA fragments by micrococcal nuclease fused to H2A and H3 was almost complete (Supplementary Figure S6G and 6H; 0.5–4 h), indicating that most rRNA genes were covered by nucleosomes. Consistently, upon activation of micrococcal nuclease fused to A190 the degradation of the smeary signal was considerably slow (Supplementary Figure S6E). In summary, these results imply that in *rad14Δ* most of the RNAPI were replaced by nucleosomes. Yet, upon activation of micrococcal nuclease fused to Hmo1, the degradation of smeary signals was similar for both *rad14Δ* and *WT* (compare Supplementary Figure S6F and S6B; 25S, 0.5 h and 1 h and 18S, 0.5 h). Thus, Hmo1 remained associated with rDNA following UV-induced displacement of RNAPI, even in *rad14Δ*, where a considerable amount of nucleosomes were loaded on the rDNA sequences.

## DISCUSSION

At least four models are proposed to describe the fate of an arrested RNAPII at a CPD, it could: (i) transcribe through the lesion; (ii) remain at the damaged site and undergo conformational changes to allow repair (30); (iii) be moved from the damaged site by reverse translocation without separating from the DNA strand; or (iv) be released from the DNA. In contrast to our current knowledge on RNAPII, very little is known about the fate of RNAPI on the damaged TS of rRNA genes, but an *in vitro* study suggested that both RNAPII and RNAPI blocked at CPDs could be released (31). To investigate the fate of RNAPI on damaged rRNA genes *in vivo*, we exploited the yeast rDNA locus where active rRNA genes are transcribed at very high rate, are loaded with RNAPI and are largely devoid of nucleosomes. After irradiation, analyses using T4 endo V nuclease showed that the likely position of the first CPD (e.g. where most rRNA genes, ∼87.3%, had at least 1 CPD in the TS) corresponded to ∼2.96 kb downstream of the transcription start site. In *WT* (*NER^+^*) cells, RNAPI occupancy behind this region dropped to ∼20% within the first 30 min after irradiation. After 4 h, most of the RNAPI occupancy was restored over the entire transcribed region, which correlated with the majority of rDNA being repaired (19) and with recovery of transcription that was measured by the TRO assay. Conversely in NER-deficient *rad14Δ* cells, RNAPI occupancy dropped to values near to experimental background level and there was no recovery of transcription even after 4 h. Similar drop in RNAPI occupancy were observed in a proteasome mutant strain, suggesting that the proteasome was not involved in displacing RNAPI from the damaged TS, at least in yeast. In addition, although this study focuses on the effect of total DNA damage induced by UV light, we like to mention that most of UV-induced DNA lesions are CPDs (∼75%) and that the 6-4PPs represent only ∼20% of the damage. Because both types of lesions block RNA polymerases (32), and similar amounts of CPDs were formed in the active and inactive rDNA chromatin structures (19), it is likely that most displacement of RNAP-I reported in our study was caused by CPDs. Although there are evidences that cells repair DNA lesions without displacing stalled RNAPII, here we provide good indications that RNAPI dissociated from UV-damaged rDNA. The different fates of RNAPII and RNAPI at CPDs could result from their intrinsic characteristics: whereas few RNAPII were found per transcription unit (33), rRNA genes are highly transcribed and densely covered by RNAPI (one RNAPI every ∼130 bp for yeast rDNA). Under these circumstances, dissociation of an arrested RNAPI at a CPD could result from the collision with the next approaching polymerase. This interpretation is suggested by the *in vitro* study of trailing (elongating) and leading (stalled) T7 RNA polymerases (T7 RNAP), which also transcribe DNA at very high rate (34). The collision between polymerases caused the release of the leading T7 RNAP while the trailing complex continued elongation to the stall site.

Before the likely position of the first CPD, the rDNA was occupied by elongating RNAPI throughout the incubation time following UV irradiation, in both *WT-* and NER-deficient mutants. These results were obtained by ChIP of TAP tagged RNAPI and completed by micrographs obtained from Miller’s spreads showing the rDNA/RNAPI/rRNA ternary complexes. Moreover, some accumulation (∼1.5 to 2 times) of RNAPI was observed at the rDNA 5′-end, which prompted us to monitor the presence of RNAPI transcription factors before and after UV irradiation. It is proposed that UAF recruits CF because is bound to UE of both inactive and active rRNA genes (25,26), whereas CF is only bound to active rRNA gene promoters. Accordingly, the presence of UAF remained constant during the entire incubation time following irradiation and CF binding increased ∼2 to 3 times, closely correlating with the accumulation of RNAPI at the 5′-end.

**Figure 7.**
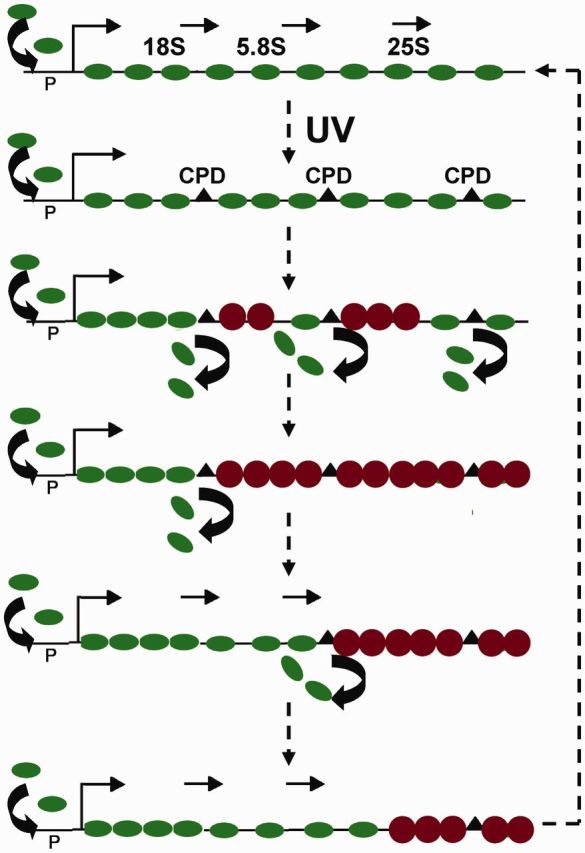
Displaced RNAPI are replaced by nucleosomes. Ovals: RNAPI, circles: nucleosomes, triangles: CPDs, P: promoter, thin arrows: initiation and direction of transcription.

In rRNA, genes transcription and open chromatin are intimately connected. After UV irradiation, psoralen cross-linking indicated closing of rDNA chromatin beyond the likely position of the first CPD. Furthermore, closing occurred in the absence of NER; that is in *rad14Δ* cells. This excluded the possibility that changes in psoralen cross-linking were caused by the presence of the NER complex itself. In fact, Rad14 participates in a very early step of the NER pathway and is required for targeting the repair complex to the damaged site (35). Additionally, the data obtained by the combination of ChEC and psoralen cross-linking clearly indicated that closing of rDNA chromatin, on what were the active genes before UV irradiation, resulted from loading of nucleosomes. However, they retained Hmo1 as signature of active rRNA genes. Therefore we suggest that as NER proceeded and transcription resumed, reopening of rDNA chromatin resulted from the replacement of nucleosomes by advancing RNAPI from the 5′-end, which might be facilitated by the presence of Hmo1. In summary, the combination of data presented here put forward a sequence of events whereby after UV irradiation there was continuous RNAPI transcription initiation, followed by elongation to the first CPD present in the TS where RNAPI dissociated. The ternary complexes resulting from transcription re-initiation and elongation prevented the deposition of nucleosomes. A different scenario took place between two CPDs, where most of the RNAPI (but not Hmo1) dissociated and were replaced by nucleosomes. After repair, elongating RNAPI restored the nonnucleosomal structure, starting at the 5′-end and sequentially spreading throughout the coding region (Figure 7). Analogously, it was shown that the replication fork entering upstream of transcriptionally active (nonnucleosomal) rRNA genes generated two newly replicated coding regions regularly packaged into nucleosomes (36). Regeneration of nonnucleosomal rDNA occurred after replication and was mediated by transcription initiation and RNAPI advancing through the template (11,36).

The efficiency of DNA repair is restricted by the presence of nucleosomes (37,38) and NER, like photolyase, repaired UV-induced photoproducts in nonnucleosomal rDNA faster than in nucleosomal rDNA (19,39). For instance, in an early study we found that inactive rRNA genes were only repaired by GGR, whereas active rRNA genes were repaired by both TCR and GGR (22). Since during the early repair times the band corresponding to active rDNA chromatin was no longer evident and was replaced by a smear, which represented partially inactivated rRNA genes, we suggest that GGR repairs patches of inactivated chromatin within active rRNA genes, and speculate that at least two processes can occur simultaneously in different rDNA regions of active rRNA genes: CPDs are processed by TCR when paused RNAPI are recognized before being released from the TS or, alternatively, paused RNAPI are released before initiation of TCR and nucleosomes are loaded, inactivating portions of rRNA genes that are repaired by GGR.

## SUPPLEMENTARY DATA

Supplementary Data are available at NAR Online, including [40–43].

## FUNDING

This work was supported by Natural Sciences and Engineering Research Council of Canada. Our collaboration was supported by the Bavarian State Chancellery (Bayerisch-Französisches Hochschulzentrum) (to J.G.) and the Ministère des Relations Internationales du Québec (to A.C. M.T., R.C. and A.C.) designed research; M.T. and R.C. did most of the experiments, R.C. and M.W. performed ChEC and G.L. provided initial help; A.C. and J.G. wrote the article. Funding for open access charge: CRSNG.

*Conflict of interest statement*. None declared.

## Supplementary Material

Supplementary Data
