# Supplementary material for: UV light-induced DNA lesions cause dissociation of yeast RNA polymerases-I and establishment of a specialized chromatin structure at rRNA genes
Source: Nucleic Acids Res. 2013 Oct 4;42(1):380–95. doi: 10.1093/nar/gkt871 (PMC3874186; doi:10.1093/nar/gkt871)
Supplement: Supplementary Data [file supp_42_1_380__index.html]

UV light-induced DNA lesions cause dissociation of yeast RNA polymerases-I and establishment of a specialized chromatin structure at rRNA genes — UV light-induced DNA lesions cause dissociation of yeast RNA polymerases-I and establishment of a specialized chromatin structure at rRNA genes — Supplementary Data 

# UV light-induced DNA lesions cause dissociation of yeast RNA polymerases-I and establishment of a specialized chromatin structure at rRNA genes

## Supplementary Data

files

**Files in this Data Supplement:**

- Supplementary Data - zip file
