# Supplementary material for: UV light-induced DNA lesions cause dissociation of yeast RNA polymerases-I and establishment of a specialized chromatin structure at rRNA genes
Source: Nucleic Acids Res. 2013 Oct 4;42(1):380–95. doi: 10.1093/nar/gkt871 (PMC3874186; doi:10.1093/nar/gkt871)
Supplement: Supplementary Data [file supp_gkt871_suppl_data.zip › nar-00638-d-2013-File016.pptx]

## Slide 1
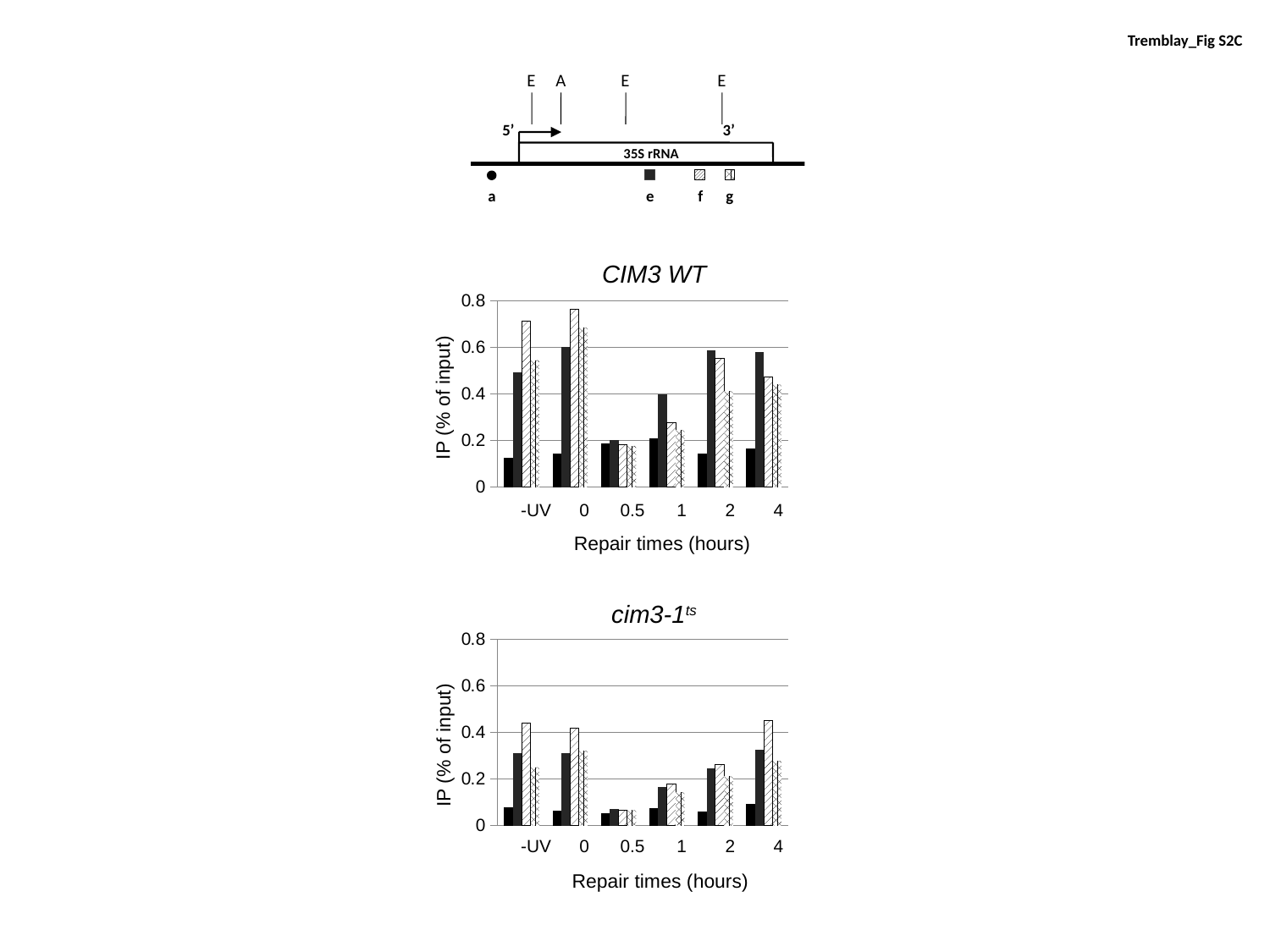

Tremblay_Fig S2C
E
A
E
E
5’ 3’
35S rRNA
f
g
a
e
CIM3 WT
### Chart
| Category | NTS2 | EcoRIB | EcoRIA | 25Sb |
|---|---|---|---|---|IP (% of input)
-UV
0
0.5
1
2
4
Repair times (hours)
cim3-1ts
### Chart
| Category | NTS2 | EcoRIB | EcoRIA | 25Sb |
|---|---|---|---|---|IP (% of input)
-UV
0
0.5
1
2
4
Repair times (hours)
