# Supplementary material for: UV light-induced DNA lesions cause dissociation of yeast RNA polymerases-I and establishment of a specialized chromatin structure at rRNA genes
Source: Nucleic Acids Res. 2013 Oct 4;42(1):380–95. doi: 10.1093/nar/gkt871 (PMC3874186; doi:10.1093/nar/gkt871)
Supplement: Supplementary Data [file supp_gkt871_suppl_data.zip › nar-00638-d-2013-File017.pptx]

## Slide 1
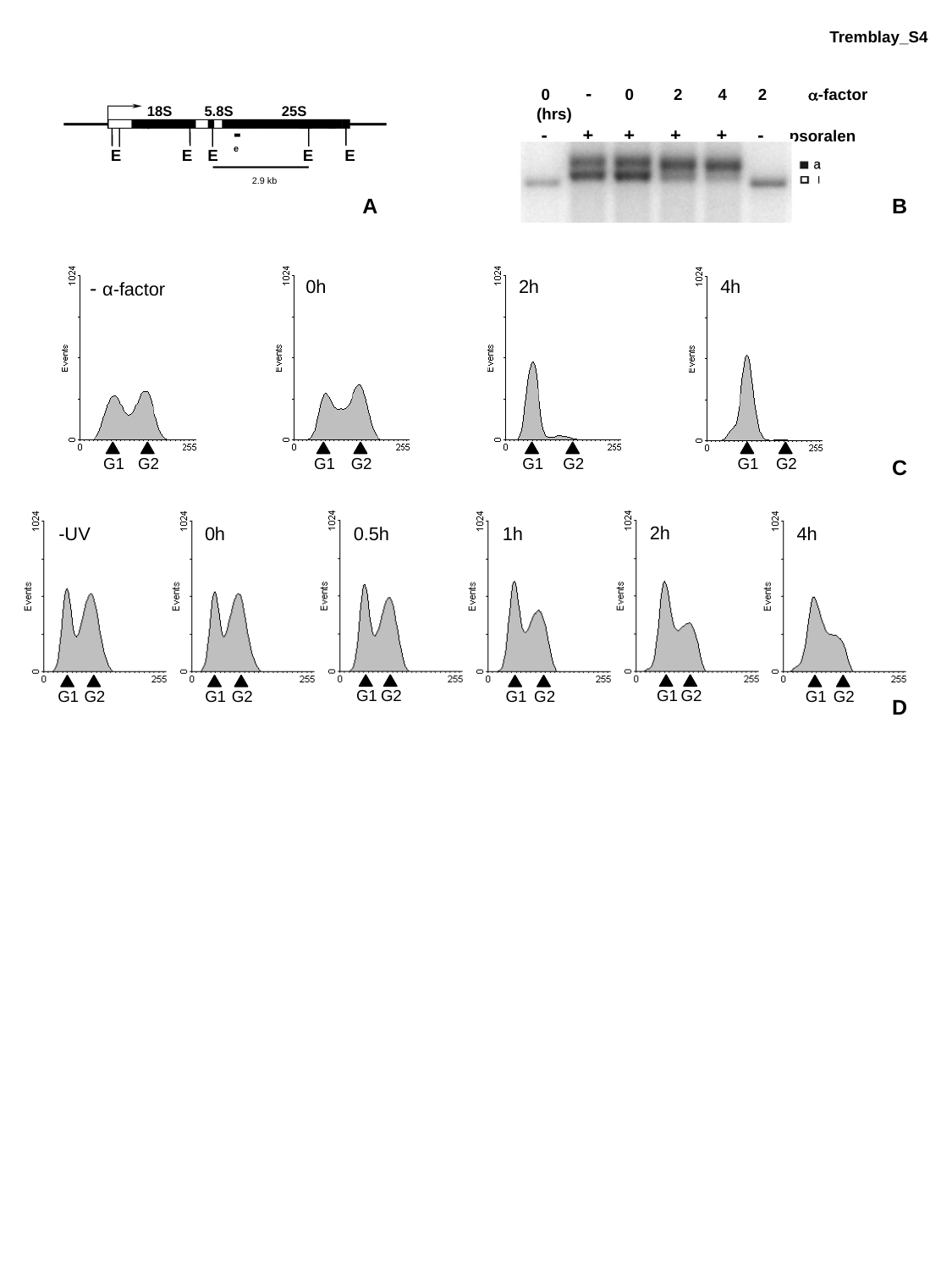

Tremblay_S4
 0 - 0 2 4 2 a-factor (hrs)
 - + + + + - psoralen
18S 5.8S 25S
e
E
E
E
E
E
2.9 kb
A
B
- α-factor
G1
G2
0h
G1
G2
2h
G1
G2
4h
G1
G2
C
2h
-UV
0h
0.5h
1h
4h
G1
G2
G1
G2
G1
G2
G1
G2
G1
G2
G1
G2
D
