# Supplementary material for: UV light-induced DNA lesions cause dissociation of yeast RNA polymerases-I and establishment of a specialized chromatin structure at rRNA genes
Source: Nucleic Acids Res. 2013 Oct 4;42(1):380–95. doi: 10.1093/nar/gkt871 (PMC3874186; doi:10.1093/nar/gkt871)
Supplement: Supplementary Data [file supp_gkt871_suppl_data.zip › nar-00638-d-2013-File018.pptx]

## Slide 1
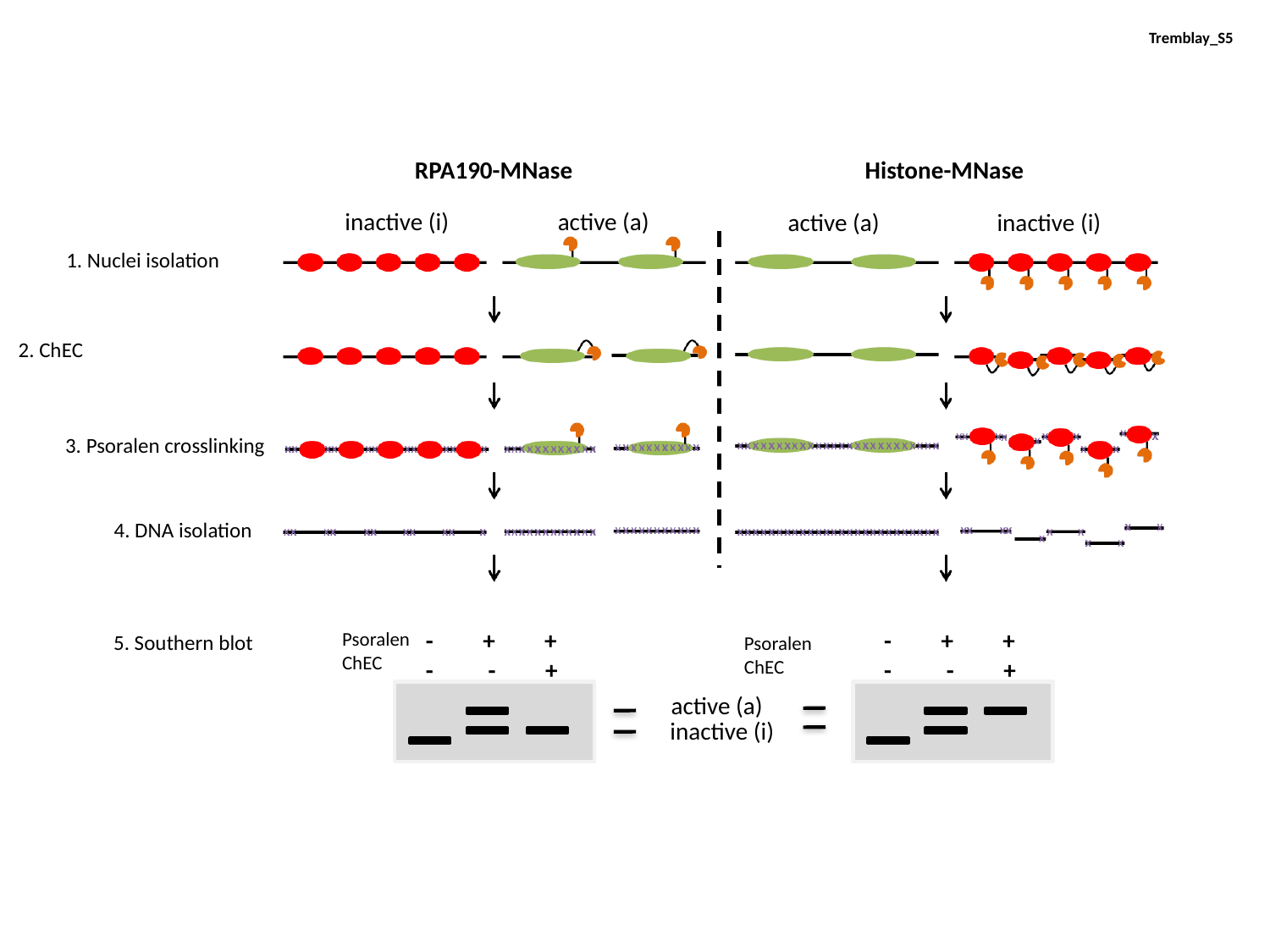

Tremblay_S5
RPA190-MNase
Histone-MNase
inactive (i)
active (a)
active (a)
inactive (i)
1. Nuclei isolation
2. ChEC
3. Psoralen crosslinking
4. DNA isolation
- + +
- - +
- + +
- - +
Psoralen
ChEC
5. Southern blot
Psoralen
ChEC
active (a)
inactive (i)
