# Supplementary material for: UV light-induced DNA lesions cause dissociation of yeast RNA polymerases-I and establishment of a specialized chromatin structure at rRNA genes
Source: Nucleic Acids Res. 2013 Oct 4;42(1):380–95. doi: 10.1093/nar/gkt871 (PMC3874186; doi:10.1093/nar/gkt871)
Supplement: Supplementary Data [file supp_gkt871_suppl_data.zip › nar-00638-d-2013-File011.docx]

**SUPPLEMENTAL INFORMATION**

**Table S1** Yeast strains used in this study.

Figure S1 Average CPD yields, and hotspots for CPD formation in the rRNA gene 5’-end and contiguous sequences (related to Figure 1). (A) Map of the 18S portion of the rRNA gene. The 5’-end and direction of transcription are shown, together with the restriction sites and their positions from the transcription initiation site (+1). The labels 1 to 10 mark the 10 hotspot-regions for CPD formation detected downstream of the 5’-end (see B). The labels ‘c’ and ‘d’ represent 2 of the amplicons used in the ChIP assay (Figure 1), and are shown here as references. The black circles represent the two 25 nucleotides long oligomers, that are complementary to the transcribed strand (TS), used for indirect end-labeling for the *Sma*I-*Apa*I and *Apa*I-*Apa*I fragments; the first nucleotide of the sequence corresponding to the positions -208 (*Sma*I site) and +1336 (*Apa*I site), respectively. (B) Yeast cells, carrying the tandem affinity purification (TAP) tagged RPA190 subunit of RNAPI (Table S1), were treated with a dose of 180 J/m^2^. DNA was isolated from cells that were harvested before (-UV) or soon after (+UV) irradiation, digested with *Sma*I and *Apa*I and mock treated or treated with *T4 endo V* (denoted by – and +, respectively), which cleaves specifically at CPD sites. DNA strands were separated on 1% alkaline agarose gels and transferred to Hybond-XL membrane (GE-Healthcare). Upper panel: indirect end labeling for the proximal 1.39 kb *Sma*I-*Apa*I fragment; lower panel: indirect end labeling for the distal 1.41 kb *Apa*I-*Apa*I fragment. Size marker (M): 1.5, 1.2, 1.0, 0.9, 0.8, 0.7, 0.6, 0.5, 0.4, 0.3, 0.2 and 0.1 kb. Labels 1 to 10 denote the hotspots for CPD formation.

Quantification of CPDs was done on data from phosphorimages using ImageQuant software (GE-Healthcare). CPD per DNA fragment were determined as following: the volume (V) of each band (± UV and ± T4 endo V) was corrected by subtracting its average background, which is the sum of equal signal volumes measured above and below the band and divided by 2. Using the corrected volumes, the following equation was applied: CPDs / DNA fragment = ln (V-T4endoV / V+T4endoV). The percentage of undamaged fragments was obtained by calculating the ratio V+T4endoV / V-T4endoV. About 5.0 CPDs were induced in the TS of the rDNA coding region (6.86 kb), ~1.0 ± 0.2 CPDs were induced in the 1.39 kb *Sma*I-*Apa*I fragment and ~1.1 ± 0.05 CPDs in the 1.41 kb *Apa*I fragment (± 1 SD of 3 independent experiments). These measurements correspond to ~0.7 and ~0.8 CPDs per kb, respectively. DNA length affects the frequency at which CPDs form and ~0.6 CPDs are expected in the 0.86 kb *Eco*RI-*Apa*I fragment. Consequently, on average, ~87.3% of the rRNA genes have at least 1 CPD in the 2.96 kb fragment, ~61.3% of the rRNA genes have at least 1 CPD in the 1.39 kb fragment and ~37.9% of the rRNA genes have at least 1 CPD in the 0.86 kb fragment.

**Figure S2** To determine if the proteasome is involved in the displacement of RNAPI from the UV damaged rDNA transcribed strand (TS) in a *WT* (*NER^+^ RAD14*) strain we used a temperature sensitive (*ts*) mutant strain for the *CIM3/SUG1* gene, which codes for an essential protein of the 19S proteasome regulatory subunit (43). (**A**) Rrn3p-degradation is proteasome dependent. To verify that shifting the temperature to 37^o^C inhibited the activity of the proteasome, we followed the stability of Rrn3p as previously described (42). *CIM3* *WT* and *cim3-1^ts^* yeast strains expressing (3x)HA-tagged Rrn3p were grown in YPD at 24^o^C to mid-log phase. Cycloheximide (CHX) was added to 100μg/ml final concentration, both cultures were shifted to 37^o^C (t = 0 min) and kept at the non-permissive temperature for up to 120 min. At the indicated time points, total proteins were isolated by resuspending 2.10^7^ cells in 150 μl of 1.85 M NaOH and 7.5% β-mercaptoethanol and then kept on ice for 15 min. After addition of 150 μl of 55% TCA, samples were incubated for 10 min on ice followed by centrifugation (18,000 g, 10 min, 4°C). 200 μl of storage buffer (62.5 mM Tris [pH 8.8], 2% SDS, 10% glycerol) were added to each pellet. Protein concentrations were measured by the Bradford method and 20 μg of each sample were diluted in Laemmli buffer (62.5 mM Tris pH 8.8, 2% SDS, 5% β-mercaptoethanol, 10% glycerol, 0.01% bromophenol blue). Electrophoresis (10% SDS-PAGE) and western blotting were done as standard procedure. After staining with Ponceau red (lower panel) to control for loading, filter membranes were hybridized with rat monoclonal antibodies against HA (3F10, Roche) (upper panel). The left panel shows the time course for degradation of Rrn3p by the proteasome (*CIM3 WT*) and the results are as previously described (42). The right panel indicates that the activity of the proteasome was inhibited upon shifting the *cim3-1^ts^* strain to 37^o^C. (**B**) Stability of the RNAPI subunit Rpa43 before and after UV irradiation. *CIM3 WT* and *cim3-1^ts^* yeast strains (*NER^+^*) expressing TAP-tagged Rpa43 were grown in YPD at 24^o^C to mid-log phase. Cultures were mock treated (- CHX) or treated with cycloheximide (+ CHX) as described in (A) and incubated at 37^o^C for 30 min. Thereafter, cells were irradiated (+ UV) and incubated for different repair times (0, 0.5, 1, 2 and 4 hrs). As control, *CIM3* *WT* and *cim3-1^ts^* strains were incubated for up to 4 h in the presence of CHX but were not UV irradiated [+ CHX; no UV, where C (controls) are samples from cells that were not treated with cycloheximide]. Total protein isolation was done as in (A) and the filter membranes were hybridized with rabbit polyclonal antibodies against protein A to detect the TAP tagged Rpa43p (p3775, Sigma) and with rat monoclonal antibodies (YOL1/34) to detect tubulin (ab6161, Abcam) (loading control). Left upper panel: (- CHX, + UV) shows the steady state of Rpa43p after UV irradiation. Left middle panel: (+ CHX, no UV) shows that the Rpa43p subunit of RNAPI is a stable protein compared to Rrn3p (see Figure S2A). Left lower panel: (+ CHX, + UV) indicates that UV irradiation does not induce major changes in Rpa43p stability. Right panels: show that results obtained with the *cim3-1^ts^* are very similar to those obtained with *CIM3 WT*. (**C**) The proteasome does not participate in the displacement of RNAPI upon UV irradiation. To determine if the proteasome is required for the displacement of RNAPI from the damaged TS (see Figures 1 and 3), the occupancy of RNAPI was mapped between the central and 3’-end region of the 35S rRNA-coding sequence in the *CIM3 WT* (*NER^+^*) and *cim3-1^ts^* mutant (*NER^+^*). *CIM3* *WT* and *cim3-1^ts^* yeast strains expressing TAP tagged Rpa43p were grown in YPD at 24^o^C to mid-log phase. After temperature shift cells were kept at 37^o^C for 30 min, UV irradiated and incubated for different times to allow repair. Chromatin-immunoprecipitations (ChIP) on formaldehyde crosslinked cells were performed with IgG coated beads that bind TAP tagged Rpa43p. Real time amplification of co-precipitated DNA was done using a series of amplicons (upper panel; a: control, e, f and g). In *CIM3* *WT* drop in RNAPI occupancy was observed between 0.5 and 1 hour after irradiation, and after 2 to 4 hours most of the RNAPI occupancy was restored (middle panel). Similar results were obtained with the *cim3-1^ts^* mutant (lower panel), indicating that RNAPI displacement occurred in absence of proteasome activity (compare with Figure 1B).

**Figure S3** Electron microscopy of chromatin prepared by Miller’s spreading (related to Figure 3). Chromatin spreads were prepared as described in Methods. We note that stationary cells and cell cycle arrested cells present changes in cell wall composition that make them more resistant to zymolase digestion. Consequently, chromatin spreading is more difficult under these conditions. After visual analyses of several grids obtained from UV irradiated *rad14Δ* cells, we observed only two near full-length transcription units (see *rad14Δ* 2h). Although these were an exception, their lengths were included in the histogram (Figure 3B). Upper panels: micrographs of chromatin regions containing rDNA transcription units. Bars correspond to 500 nm. Bottom panels: traces (blue lines) of chosen rDNA transcription units that were used for quantifications (Figure 3B). The lengths of transcription units were measured using ImageJ software (<http://rsbweb.nih.gov/ij/>), whereby the lines were compared to the internal scale bars.

**Figure S4** Opening of rRNA genes in G1 arrested cells (supplementary results). (A) Map of the yeast 35S rRNA gene with the 18S, 5.8S and 25S coding regions. The probe ‘e’ (same as the amplicon in Figure 1A) and the *Eco*RI restriction site (E) are shown. (B) As control, the dynamic of rDNA chromatin in cells that were synchronized in G1 by addition of α-factor were analyzed by psoralen crosslinking. Nuclei were isolated from aliquots of cells (JS306- A190MN-*bar1Δ*) that were mock treated (- α-factor) or treated with α-factor and then collected at different incubation times (0, 2 and 4 h). After psoralen crosslinking DNA was extracted, digested with *Eco*RI and separated on 1% native agarose gels. After blotting, the filter membranes were hybridized with ^32^P end-labeled oligonucleotide ‘e’; a: active and i: inactive rDNA chromatin. (C) In parallel, cell aliquots were prepared for Flow Cytometry. The results show that most of the rRNA genes opened when cells arrested in G1 [compare figures (B) and (C)]. (D) Cells (JS311), non-irradiated (-UV) or UV irradiated (0 h), were incubated for different repair times (0.5 to 4 h), and prepared for Flow Cytometry. The results show that at 4 h there was some accumulation of cells arrested in G1/S, which can explain the additional opening of rRNA genes after 4 h repair (see Figures 5 and 6).

**Figure S5** Schematic representation of the combined ChEC and psoralen crosslinking assay (supplementary information). Green and red ovals represent RNAPI and nucleosomes, repectively; the orange semicircles represent micrococcal nuclease (MNase) tags. The purple crosses represent psoralen crosslinks. Left: MNase is fused to a subunit of RNAPI (component of the a-band), and right MNase is fused to a histone protein (component of the i-band). 1: Nuclei are isolated from yeast expressing single MNase fused proteins and, 2: incubated in the presence of Ca^2+^ to induce cleavage (ChEC). Thereafter, 3: nuclei are photo-crosslinked with psoralen and, 4: the DNA is isolated and digested with the appropriate restriction enzymes, separated by agarose gel electrophoresis and, 5: analyzed by Southern blot. The examples show three-lanes filter membranes representing the control (no ChEC, no psoralen crosslinking), separation of active and inactive rDNA by psoralen crosslinking (no ChEC), and analysis of rDNA protein content by ChEC for active (left) and inactive rDNA (right).

**Figure S6** Closing and re-opening of rRNA genes chromatin resulted from nucleosomes loading and unloading (related to Figure 6A-D). Yeast *WT* (*NER^+^*) expressing the RNAPI subunit A190-, Hmo1-, H2A- or H3- micrococcal nuclease fusion proteins, non-irradiated (-UV) or UV irradiated (0h), were incubated for different times to allow repair (0.5h to 4h). Nuclei were isolated and ChEC was initiated by adding CaCl_2_ to the nuclei suspensions. DNA cleavage was allowed to proceed for various times as indicated by ‘min ChEC’, where ‘0’ is the control (CaCl_2_ mock treated). After addition of EDTA containing buffer to stop the nuclease activity, psoralen crosslinking, DNA extraction and *Eco*RI restriction enzyme digestion (see Figure 5A), the DNA samples were separated on 1% native agarose gels and blotted to filter membranes that were probed to detect the 2.23 and 2.9 kbp *Eco*RI fragments containing portions of the 18S and 25S regions, respectively. Symbols are: “a” for active and “i” for inactive rDNA chromatin.

Closing of rRNA genes chromatin in NER deficient cells after UV irradiation resulted from nucleosomes loading (related to Figure 6E-H). Yeast *rad14Δ* expressing the RNAPI subunit A190-, Hmo1-, H2A- or H3- micrococcal nuclease fusion proteins, non-irradiated (-UV) or UV irradiated (0h), were incubated for different times (0.5h to 4h). ChEC and DNA analyses were done as described in Figure S6A-D. Filter membranes were probed to detect the 2.23 and 2.9 kbp *Eco*RI fragments (E, F and G) containing portions of the 18S and 25S regions, respectively, or the 2.23, 2.9 and 2.44 kbp fragment (H) containing the IS. Symbols are: “a” for active and “i” for inactive rDNA chromatin.
