# Supplementary material for: UV light-induced DNA lesions cause dissociation of yeast RNA polymerases-I and establishment of a specialized chromatin structure at rRNA genes
Source: Nucleic Acids Res. 2013 Oct 4;42(1):380–95. doi: 10.1093/nar/gkt871 (PMC3874186; doi:10.1093/nar/gkt871)
Supplement: Supplementary Data [file supp_gkt871_suppl_data.zip › nar-00638-d-2013-File012.docx]

| *Strain* | *Parent* | *Genotype* | *Origin* |
| --- | --- | --- | --- |
| JS311 |  | MATα, his3Δ200, leu2Δ1, met15Δ0, trp1Δ63, ura3-167, RDN1::Ty1-MET15, mURA3/HIS3 | (40) |
| JS311-A190MN | JS311 | JS311 made RPA190-MNase-3xHA::KanMX6 | This study |
| JS311-A190MN  *Rad14Δ* | JS311-A190MN | JS311-A190MN made rad14Δ::LEU2 | This study |
| JS311-HMO1MN | JS311 | JS311 made HMO1-MNase-3xHA::KanMX6 | This study |
| JS311-HMO1MN  *rad14Δ* | JS311-HMO1MN | JS311-HMO1MN made rad14Δ::LEU2 | This study |
| JS311-H2AMN | JS311 | JS311 made HTA1-MNase-3xHA::KanMX6 | This study |
| JS311-H2AMN  *rad14Δ* | JS311-H2AMN | JS311-H2AMN made rad14Δ::LEU2 | This study |
| JS311-H3MN | JS311 | JS311 made HHT1-MNase-3xHA::KanMX6 | This study |
| JS311-H3MN  *rad14Δ* | JS311-H3MN | JS311-H3MN made rad14Δ::LEU2 | This study |
| JS306-A190MN | JS306 | MATa, his3Δ200, leu2Δ1, met15Δ0, trp1Δ63, ura3-167, RDN1::Ty1-MET15, mURA3/HIS3, RPA190-MNase-3xHA::KanMX6 | This study |
| JS306-A190MN-*bar1Δ* | JS306-A190MN | JS306-A190MN made bar1Δ::TRP1 | This study |
| 190TAP |  | MATa, ade2, arg4, leu2, trp1-289,ura3-52, RPA190::TAP-K.I.URA3 | (41) |
| *190TAP-rad14*Δ | 190TAP | 190TAP made rad14Δ::KanMX | This study |
| y618 | NOY505 | MATa; ade2-1; ura3-1; trp1-1; leu2-3,112; his3-11; can1-100; UAF30-MNase-3xHA::KanMX6 | (12) |
| y1141 | NOY505 | MATa; ade2-1; ura3-1; trp1-1; leu2-3,112; his3-11; can1-100; RRN11-MNase-3xHA::KanMX6 | (12) |
| y657 | YWO365 | MATa ; ura3-52 ; his3-Δ200 ; leu2- Δ1 ; trp1-Δ63 ; lys2-801 ; ade2-101 ; prc1-1 ; RPA43-TAP(URA3) ; RRN3-HA3(HIS) | (42) |
| y660 | YWO366 | MATa ; ura3-52 ; his3-Δ200 ; leu2-Δ1 ; prc1-1 ; cim3-1 ; RPA43-TAP(URA3) ; RRN3-HA3(HIS) | (42) |
