# Supplementary material for: UV light-induced DNA lesions cause dissociation of yeast RNA polymerases-I and establishment of a specialized chromatin structure at rRNA genes
Source: Nucleic Acids Res. 2013 Oct 4;42(1):380–95. doi: 10.1093/nar/gkt871 (PMC3874186; doi:10.1093/nar/gkt871)
Supplement: Supplementary Data [file supp_gkt871_suppl_data.zip › nar-00638-d-2013-File015.pptx]

## Slide 1
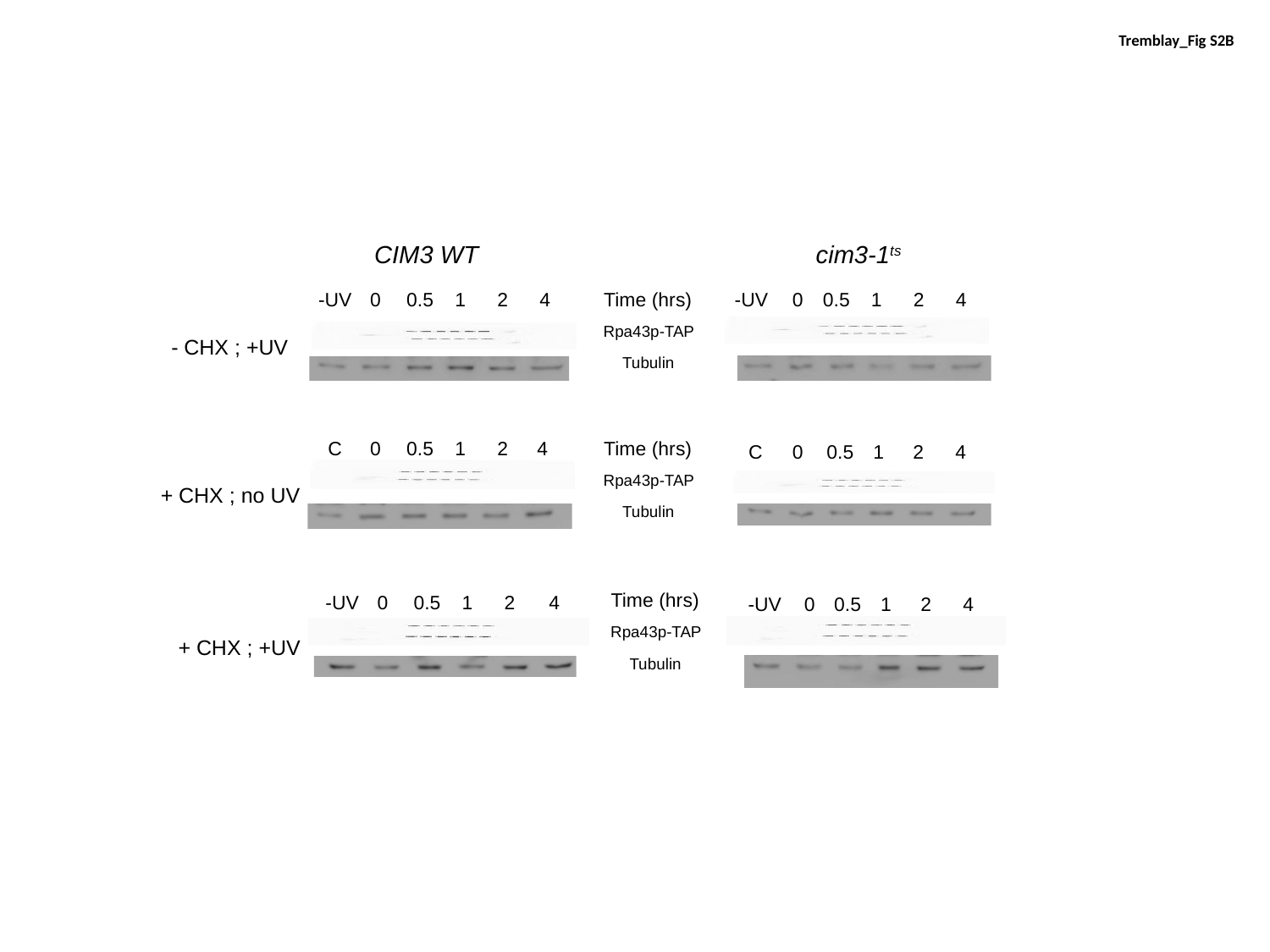

Tremblay_Fig S2B
CIM3 WT
cim3-1ts
-UV
0
0.5
1
2
4
Time (hrs)
Rpa43p-TAP
Tubulin
-UV
0
0.5
1
2
4
- CHX ; +UV
C
0
0.5
1
2
4
Time (hrs)
Rpa43p-TAP
Tubulin
 C
0
0.5
1
2
4
+ CHX ; no UV
Time (hrs)
Rpa43p-TAP
Tubulin
-UV
0
0.5
1
2
4
-UV
0
0.5
1
2
4
+ CHX ; +UV
