# Supplementary figures and images for: UV light-induced DNA lesions cause dissociation of yeast RNA polymerases-I and establishment of a specialized chromatin structure at rRNA genes
Source: Nucleic Acids Res. 2013 Oct 4;42(1):380–95. doi: 10.1093/nar/gkt871 (PMC3874186; doi:10.1093/nar/gkt871)

## Slide 1
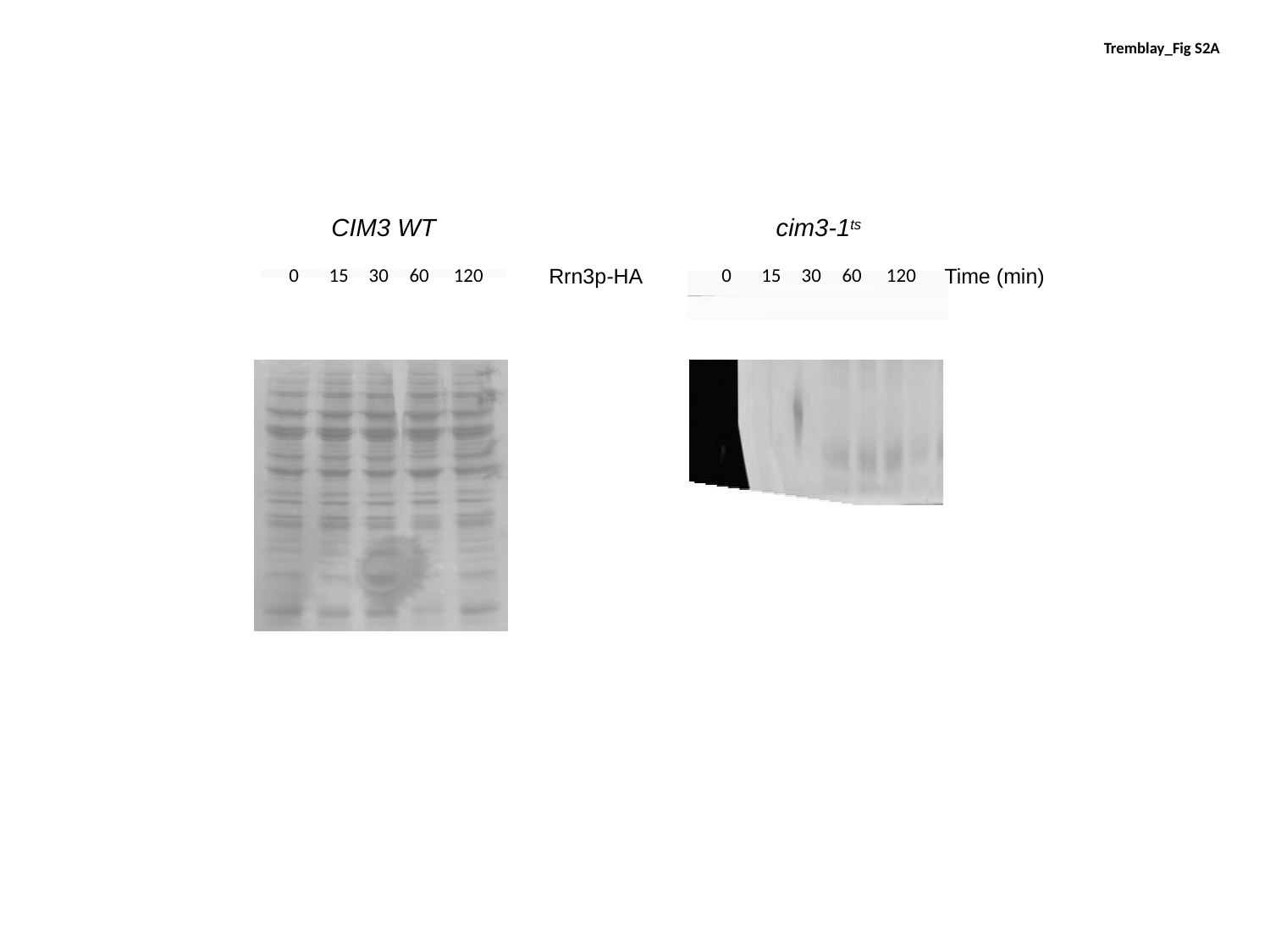

Tremblay_Fig S2A
CIM3 WT
cim3-1ts
0
15
30
60
120
Rrn3p-HA
0
15
30
60
120
Time (min)

Supplement: Supplementary Data [file supp_gkt871_suppl_data.zip › nar-00638-d-2013-File014.pptx]
